# Value of preoperative evaluation of FEV_1_ in patients with destroyed lung undergoing pneumonectomy - a 20-year real-world study

**DOI:** 10.1186/s12890-024-02858-5

**Published:** 2024-01-17

**Authors:** Wenbo Li, Jing Zhao, Changfan Gong, Ran Zhou, Dongjie Yan, Hongyun Ruan, Fangchao Liu

**Affiliations:** 1 Faculty of Health and Life Science, The University of Exeter, Exeter, UK; 2 Department of Anesthesia, Beijing Chest Hospital, Capital Medical University, Beijing Tuberculosis and Thoracic Tumor Research Institute, Beijing, P. R. China; 3 Department of Thoracic Surgery, Beijing Chest Hospital, Capital Medical University, Beijing Tuberculosis and Thoracic Tumor Research Institute, No 9, Bei guan Street, Tong Zhou District, Beijing, 101149 P. R. China; 4 Department of General Medicine, Qingdao Chest Hospital, Qingdao, P. R. China; 5 Department of Cellular and Molecular Biology, Beijing Chest Hospital, Capital Medical University, Beijing Tuberculosis and Thoracic Tumor Research Institute, No 9, Bei guan Street, Tong Zhou District, Beijing, 101149 P. R. China; 6 Department of Science and Technology, Beijing Chest Hospital, Capital Medical University, Beijing Tuberculosis and Thoracic Tumor Research Institute, No 9, Bei guan Street, Tong Zhou District, Beijing, 101149 P. R. China

**Keywords:** Destroyed lung, Forced expiratory volume in one second (FEV_1_), Pneumonectomy, Prognosis, Mortality

## Abstract

**Background:**

Clinical guidelines recommend a preoperative forced expiratory volume in one second (FEV_1_) of > 2 L as an indication for left or right pneumonectomy. This study compares the safety and long-term prognosis of pneumonectomy for destroyed lung (DL) patients with FEV_1_ ≤ 2 L or > 2 L.

**Methods:**

A total of 123 DL patients who underwent pneumonectomy between November 2002 and February 2023 at the Department of Thoracic Surgery, Beijing Chest Hospital were included. Patients were sorted into two groups: the FEV_1_ > 2 L group (*n* = 30) or the FEV_1_ ≤ 2 L group (*n* = 96). Clinical characteristics and rates of mortality, complications within 30 days after surgery, long-term mortality, occurrence of residual lung infection/tuberculosis (TB), bronchopleural fistula/empyema, readmission by last follow-up visit, and modified Medical Research Council (mMRC) dyspnea scores were compared between groups.

**Results:**

A total of 96.7% (119/123) of patients were successfully discharged, with 75.6% (93/123) in the FEV_1_ ≤ 2 L group. As compared to the FEV_1_ > 2 L group, the FEV_1_ ≤ 2 L group exhibited significantly lower proportions of males, patients with smoking histories, patients with lung cavities as revealed by chest imaging findings, and patients with lower forced vital capacity as a percentage of predicted values (FVC%pred) (*P* values of 0.001, 0.027, and 0.023, 0.003, respectively). No significant intergroup differences were observed in rates of mortality within 30 days after surgery, incidence of postoperative complications, long-term mortality, occurrence of residual lung infection/TB, bronchopleural fistula/empyema, mMRC ≥ 1 at the last follow-up visit, and postoperative readmission (*P* > 0.05).

**Conclusions:**

As most DL patients planning to undergo left/right pneumonectomy have a preoperative FEV_1_ ≤ 2 L, the procedure is generally safe with favourable short- and long-term prognoses for these patients. Consequently, the results of this study suggest that DL patient preoperative FEV_1_ > 2 L should not be utilised as an exclusion criterion for pneumonectomy.

## Introduction

Destroyed lung (DL) is a pulmonary disorder characterised by diffuse structural destruction of the lung resulting from chronic focal infection of lung tissues, as evidenced by chest CT imaging findings revealing multiple fibrotic lesions, bronchiectasis, necrosis, cavities, and calcification [1–4]. This serious condition, which can lead to almost complete loss of lung function, is more prevalent in developing countries, where approximately one-half to two-thirds of patients experience varying degrees of respiratory dysfunction [1, 5, 6]. Pathogens commonly implicated in DL causation include *Mycobacterium tuberculosis* and/or diverse fungal species, which can trigger recurrent infections that cannot be controlled with chemotherapy, as reflected by repeatedly positive sputum cultures. Such infections can escalate, leading to development of massive hemoptysis requiring surgical removal of infected tissues to save lives [7–9]. In addition, surgery can improve patients’ quality of life by removing multidrug-resistant TB (MDR-TB) bacilli and damaged tissues from the lungs to prevent infection recurrence and restore respiratory function [10–14].

Preoperative pulmonary function tests are valuable tools used to assess surgical eligibility and the optimal scope of surgical lung resection. Notably, forced expiratory volume in one second (FEV_1_) was identified by Boush et al. (1971) as a crucial preoperative evaluation benchmark for predicting patient tolerance of thoracic surgery [15]. Their findings demonstrated that for every 10% decrease in FEV_1_ value, the incidence of lung-related complications increased 1.1-fold [1, 16]. Similarly, another study of 1046 lung cancer patients conducted by Ferguson et al. [17]. pinpointed FEV_1_ as an independent predictive risk factor for perioperative mortality resulting from pulmonary complications, as consistent with results of a study conducted by Licker et al. of 1239 lung cancer patients underscoring the value of FEV_1_ in predicting complications of lung surgery [18]. Based on these findings, pulmonary surgical guidelines provided by the British Thoracic Society (BTS) and the American College of Chest Physicians (ACCP) recommend the absolute value of FEV_1_ as a screening criterion for pneumonectomy candidates, with FEV_1_ > 2.0 L signifying the procedure is suitable and feasible for patients meeting this criterion [19, 20].

Notably, our thoracic surgery department has achieved favourable clinical outcomes performing unilateral pneumonectomies in DL patients with FEV_1_ ≤ 2 L. Therefore, in this retrospective study we analysed clinical data of DL patients with FEV_1_ ≤ 2 L or FEV_1_ > 2 L who underwent pneumonectomy in our hospital from 2002 to 2023. The study aimed to assess pneumonectomy effectiveness and safety in these patient groups and investigate the clinical significance of FEV_1_ as a preoperative criterion for excluding DL patients from pneumonectomy.

## Materials and methods

### Study subjects

The study included a total of 135 patients diagnosed with DL who underwent pneumonectomy at Beijing Chest Hospital, a hospital affiliated with Capital Medical University, from November 2002 to February 2023. Among them, 12 patients who did not undergo preoperative lung function testing were excluded, leaving a total of 123 patients for the analysis. Based on preoperative forced expiratory volume in one second (FEV_1_) value, patients were sorted into two groups: the FEV_1_ > 2 L group (30 cases) and the FEV_1_ ≤ 2 L group (96 cases).

Clinical data were extracted from the hospital’s electronic medical records system. The study design adhered to the principles outlined in the Helsinki Declaration. The requirement for patient written informed consent for participation in the study was waived by the Ethics Committee of the Institutional Review Board of Beijing Chest Hospital, an affiliate of Capital Medical University (Ethics number: Clinical Research 2018 (43)).

Inclusion criteria were as follows: postoperative pathological morphology consistent with TB or inflammation, meeting surgical treatment criteria for DL patients, all of these no sign of respiratory failure, as based on preoperative arterial blood gas analysis.

Exclusion criteria were as follows: bronchial asthma, chronic obstructive pulmonary disease, interstitial lung disease, concurrent malignant tumours, chronic heart failure, chronic renal insufficiency, one or more of these.

Indications of DL patient suitability for pneumonectomy were as follows: lesions located in one lung, life-threatening massive hemoptysis, DL-related recurrent infections, MDR-TB, inability to tolerate anti-TB treatment, repeated positive results of sputum culture and/or smear for *M. tuberculosis*.

Demographic data included gender, age, body mass index (BMI), smoking history, history of alcohol use, major complications, and common comorbidities (hypertension, coronary heart disease, and diabetes). General clinical data included primary symptoms at admission and duration of the current bout of infection/TB (in months). Infections with MDR-TB, extensively drug-resistant TB (XDR-TB), and chronic aspergillosis were documented.

The modified Medical Research Council dyspnea scale (mMRC) was employed to assess dyspnea severity at time of admission then patients were sorted into two groups based on mMRC score: mMRC = 0 and mMRC ≥ 1. Preoperative auxiliary evaluations included chest CT scans, pulmonary function tests, and routine laboratory tests. Chest CT scan interpretation included the identification of spinal scoliosis and TB cavities within the lesion area. Lung function measurements were conducted using Master Screen-IOS and Master Screen-PFT instruments (Jaeger, Germany). The single-breath diffusion method was employed to determine lung diffusion capacity and predicted lung function values as based on instrument default values endorsed by the European Respiratory Society. Key indicators assessed via lung function testing included forced vital capacity (FVC), FEV_1_, total lung capacity (TLC), and diffusing capacity of the lungs for carbon monoxide (DLCO).

Surgery-related data included the following: intraoperative blood loss, duration of surgery, left or right lung resection, and performance of pleural stripping. Pneumonectomies were performed as open surgeries.

Short-term postoperative observation-based indicators included death during hospitalisation or within 30 days after discharge, postoperative complications (requiring invasive respiratory support, residual lung infection, heart failure), and median duration of postoperative complications (in days).

Follow-up face-to-face or telephone interviews were administered by a healthcare provider through June 30, 2023 to assess participant survival status, postoperative TB/pulmonary infection recurrence, respiratory function, readmission history, and postoperative treatment. Three patients died during the perioperative period. Of the 120 participants, 26 Of the initial participants (21.7% of 120) became unreachable after three unsuccessful attempts to establish contact via telephone, leading to their classification as lost to follow-up. within the perioperative period, three patients passed away. Subsequently, the follow-up was successfully completed by the remaining 94 patients, with an average age of 39.8 ± 13.6 years. This cohort comprised 28 males and 66 females.

### Quality control

Overall aims of this study included the establishment of a standardised follow-up program, a training program, and a follow-up methodology. The follow-up program design and questionnaire were reviewed and validated by senior respiratory medicine and thoracic surgery clinicians prior to follow-up program implementation under supervision of highly trained general practitioners (GPs). GPs were responsible for conducting face-to-face or telephone interviews to gather comprehensive patient clinical data following hospital discharge.

### Statistical analysis

Data analysis was conducted using the SPSS 25.0 statistical software package (SPSS Inc., Chicago, IL, USA) and included analysis of both continuous and categorical variables. Normally distributed continuous variables are expressed as mean ± standard deviation, with intergroup comparisons conducted using the t-test. Non-normally distributed continuous variables are expressed as median (25%, 75%) percentile, with intergroup comparisons conducted using the Z-test. Categorical variables are presented as composition ratios or percentages (%), with intergroup comparisons conducted using the chi-square test.

The study aimed to compare short-term mortality and postoperative complications between FEV_1_ > 2 L and FEV_1_ ≤ 2 L groups. Additionally, follow-up observations were compared between groups regarding rates of mortality, residual lung reinfection, bronchial stump fistula/empyema, and readmission, as well as postoperative proportions of patients with mMRC ≥ 1.

Binary logistic regression analysis was performed to identify correlations between independent variables (gender, age, current or past smoking status, presence of cavities on chest CT images and the continuous variable FEV_1_) and dependent variables related to clinical short- and long-term outcomes. Outcomes variables included rates of postoperative 30-day mortality, incidence of postoperative complications, long-term mortality, residual lung reinfection, TB recurrence, and bronchial stump fistula/empyema, as well as the proportion of patients with mMRC ≥ 1 at follow-up and the postoperative rehospitalisation rate. Clinically relevant statistically significant factors (*P* < 0.05) are listed in Table 1.

**Table 1:** Baseline demographics and clinical characteristics of enrolled patients

Variables	Total (*n* = 123, %)	FEV_1_ >2 L (*n* = 30, %)	FEV_1_ ≤ 2 L (*n* = 93, %)	*P* value
Sex, M	42(34.1)	18.0(60.0)	24.0(25.8)	0.001
Age group	40.1 ± 13.8	39.5 ± 14.3	40.2 ± 13.7	0.792
BMI, kg/m^2^	21.3 ± 3.1	21.0 ± 3.2	21.4 ± 3.1	0.527
Smoking history*	14(11.4)	7(23.3)	7(7.5)	0.027
Alcohol abuse	7(5.7)	3(10.0)	4(4.3)	0.360
Comorbidities				
Previous history of pulmonary tuberculosis	36(12,120)	42(9,120)	36(12,174)	0.354
Other disease^#^	22(17.9)	6(20.0)	16(17.2)	0.728
CPA	18(14.6)	3(10.0)	15(16.1)	0.392
MDR/XDR	20(16.3)	7(23.3)	13(14.0)	0.242
Chronic pulmonary symptoms				
mMRC ≥ 1	70(56.9)	14(46.7)	56(60.2)	0.193
Massive hemoptysis	20(16.3)	4(13.3)	16(17.2)	0.611
Fever	18(14.6)	5(16.7)	13(14.0)	0.720
Sputum	71(57.7)	17(56.7)	54(58.1)	0.893
Pulmonary function test				
FVC % pred	59.1 ± 12.8	65.1 ± 14.4	57.2 ± 11.6	0.003
DLCO % pred	57.2 ± 19.2	54.3 ± 13.5	58.1 ± 20.7	0.336
Chest CT				
Cavity	37(30.1)	14(46.7)	23(24.7)	0.023
Contralateral Pulmonary lesions	82(66.7)	20(66.7)	62(66.7)	1.000
Ancillary blood test				
HB, g/L	121.6 ± 15.4	125.9 ± 15.7	120.2 ± 15.2	0.079
ALB, g/L	38.7 ± 5.7	40.0 ± 7.36	38.3 ± 5.1	0.161
CRP, mg/L	8.4(2.2,29.0)	15.5(2.8,39.2)	7.4(2.0,22.2)	0.062
The operation factors				
Surgical removal of right	25(20.3)	5(16.7)	20(21.5)	0.567
Time of operation, h	152(120,180)	150(120,185)	155(120,180.0)	0.739
Mean bleeding volum, mL	700(300,1300)	800(475,2050)	600(300,1150)	0.180

Note: *, Ex or current smoker; #, Other comorbidities including 2 cases of hepatitis B, 2 cases of Ankylosing Spondylitis, 1 cases of rheumatoid, 1 cases of gastric ulcer, 2 cases of hypertension and 6 cases of coronary heart disease; In view of small sample size of patients with comorbidities, they were combined into one group for statistical analysis

Abbreviation: DL, destroyed lung; mMRC, Modified British Medical Research Council; BMI, body mass index; PFT, Pulmonary function test; CPA, chronic pulmonary aspergillosis; MDR/XDR, multidrug resistant tuberculosis/ extensively drug-resistant; HB, hemoglobin; ALB, albumin; CRP, C-reaction protein; FVC% pred, forced vital capacity of predicted value; FEV_1_ (% pred), forced expiratory volume in one second of predicted; MMV (% pred), maximal minute ventilation of predicted; DLCO (% pred), lung diffusion capacity of predicted

## Results

**Fig. 1:**
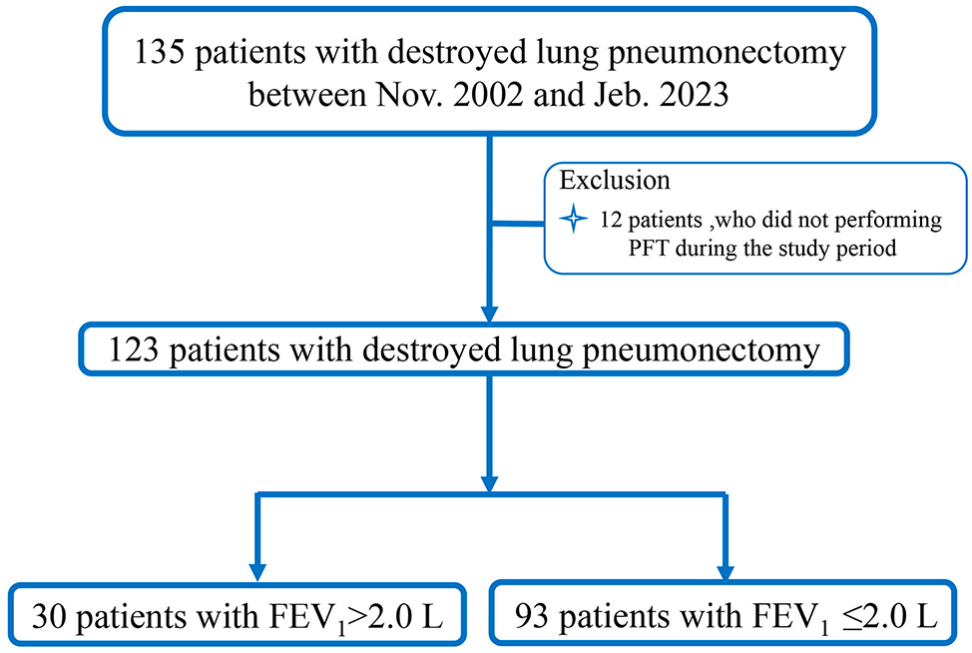
Flowchart of patients underwent pulmonary resection for damaged lung

A total of 123 patients who underwent pneumonectomy for DL were included in the study. Among them, 75.6% (93/123) had preoperative lung function FEV_1_ values of ≤ 2 L and 96.7% (119/123) were discharged without postoperative complications. The FEV_1_ > 2 L group was comprised of 30 participants, accounting for 24.4% (30/123) of patients. This group included 18 males and 12 females with an overall mean age of 44.6 ± 12.3 years. In contrast, the FEV_1_ ≤ 2 L group consisted of 93 cases, accounting for 75.6% (93/123) of patients. This group included 24 males and 69 females with an overall mean age of 40.2 ± 13.7 years (Fig. 1). Pathological examinations confirmed TB-related lung damage in 77 cases (62.6%) and identified massive hemoptysis in 20 cases (16.3%). At time of admission, 70 cases (56.9%) had mMRC scores of ≥ 1 (Table 1).

As compared to the FEV_1_ ≤ 2 L group, the FEV_1_ > 2 L group exhibited a higher proportion of males and smokers (*P* = 0.001 and *P* = 0.027, respectively), while the FEV_1_ > 2 L group exhibited a significantly higher mean FVC%pred value as compared to that of the FEV_1_ ≤ 2 L group (65.1 ± 14.4 vs. 57.2 ± 1.6, respectively, *P* = 0.003). Moreover, the proportion of patients with detectable pulmonary cavities on chest CT scans was higher in the FEV_1_ > 2 L group than in the FEV_1_ ≤ 2 L group (46.7% vs. 24.7%, respectively, *P* = 0.023) (Table 1).

Comparisons of short-term surgical outcomes revealed no significant intergroup differences in the mortality rate within 30 days after surgery/discharge or the postoperative complication rate. Similarly, comparisons of long-term outcomes through the end of the follow-up period revealed no significant intergroup differences in rates of mortality, residual lung reinfection/TB recurrence, bronchial stump fistula/empyema, mMRC score, and rehospitalisation (Table 2).

**Table 2:** Outcomes of short time and Long time follow up

Follow up	Total	FEV_1_>2 L	FEV_1_ ≤ 2 L	*P* value
**Outcomes of short time follow up**	***n*** **= 123**	***n*** **= 30(%)**	***n*** **= 90(%)**	
Death after operation ^a^	4(3.3)	0(0)	4(3.3)	0.571
Post-operative complications	33(26.8)	10(33.3)	23(24.7)	0.355
Invasive respiratory support	14(11.4)	2(6.7)	12(12.9)	0.325
Residual lung infection ^b^	14(11.4)	5(16.7)	9(9.7)	0.313
Congestive heart failure	5(4.1)	2(6.7)	3(3.2)	0.595
Duration of complications (median, IQR)	13(7,15)	1.2(0.4,3.5)	14(12,16)	0.320
**Outcomes of long time follow up**	***n*** **= 94(%)**	***n*** **= 27(%)**	***n*** **= 67(%)**	
Death	7(7.5)	3(11.1)	4(6.1)	0.410
Residual lung infection/tuberculosis	14(15.1)	5(18.5)	9(13.6)	0.557
Bronchopleural fistula/pyothorax^c^	3(3.2)	1(3.7)	2(3.0)	1.000
mMRC ≥ 1	18(19.4)	6(22.2)	12(18.2)	0.654
Aggravate and rehospitalization	11(11.8)	5(18.5)	6(9.1)	0.217

Note: a, Death after operation, during hospitalization or within 30 days of discharge; b, Residual lung infection including 5 cases of lung bacterial infections, 9 cases of Recurrence of tuberculosis; c, Bronchopleural fistula/ pyothorax including, 2 cases of Bronchopleural fistula and1 cases of pyothorax including

Abbreviation: FEV_1,_ forced expiratory volume in one second of predicted; mMRC, Modified British Medical Research Council

As shown in Table 3, results of logistic regression analysis indicated no significant differences in correlations observed between independent and dependent variables for FEV_1_ > 2 L and FEV_1_ ≤ 2 L groups in this study. Independent variables included FEV_1_ as a continuous variable and its covariates (gender, age, smoking, presence of lesions on chest CT images). Dependent variables included short-term outcomes such as mortality rates within 30 days post-surgery and postoperative complication rates, as well as long-term outcomes including rates of mortality, residual lung reinfection/TB recurrence, bronchial stump fistula/empyema, postoperative hospitalisation, and the proportion of patients with mMRC ≥ 1 during follow-up.

**Table 3:** Binary Logistic Regression Analysis of Preoperative Measured FEV_1_as a Covariate with Continuous Values and Clinical Outcome

Variables	Yes/No	FEV_1_	aOR	*P* value
Death after operation	Yes	1.4 ± 0.3	1	--
	No	1.6 ± 0.5	0.283(0.008–10.54)	0.494
Post-operative complications	Yes	1.5 ± 0.5	1	--
	No	1.6 ± 0.5	0.748(0.284–1.968)	0.557
Long-term death	Yes	1.6 ± 0.5	1	--
	No	1.6 ± 0.5	2.668(0.408–17.441)	0.306
Residual lung infection/tuberculosis	Yes	1.6 ± 0.4	1	--
	No	1.6 ± 0.5	0.434(0.122–1.539)	0.196
Bronchopleural fistula/ pyothorax	Yes	1.6 ± 0.5	1	--
	No	1.6 ± 0.5	0.822(0.080–8.479)	0.869
mMRC ≥ 1	Yes	1.5 ± 0.5		--
	No	1.5 ± 0.5	0.996 (0.319–3.108)	0.994
Aggravate and rehospitalization	Yes	1.6 ± 0.5	1	--
	No	1.6 ± 0.5	0.673(0.174–2.608)	0.567

Note: The independent variables in the binary regression mode include: age, gender, smoking history, Cavity

Abbreviation: FEV_1,_ forced expiratory volume in one second of predicted; DL, destroyed lung; mMRC, Modified British Medical Research Council

## Discussion

Surgical lung resection is an important diagnostic and therapeutic method used to treat pulmonary diseases, including DL, a severe complication of pulmonary infection [14, 21, 22]. However, surgeons view pneumonectomy as a high-risk surgical procedure [23, 24], especially when used to treat patients with pre-existing lung damage. As such, current clinical guidelines recommend a preoperative FEV_1_ value of > 2 L as an indication for surgery [19, 25, 26], with BTS/ACCP guidelines [19, 20] recommending a preoperative FEV_1_ > 2 L as an indication for pneumonectomy of lung cancer patients.

Currently, there is a notable absence of established guidelines or expert consensus regarding pneumonectomy as a DL treatment. In this study, despite the fact that 75.6% of DL patients had preoperative FEV_1_ values of ≤ 2 L, 96.7% of patients experienced successful postoperative treatment outcomes at discharge. Furthermore, no significant differences were observed between FEV_1_ > 2 L and FEV_1_ ≤ 2 L groups with regard to short-term outcome rates, including mortality within 30 days post-surgery and postoperative complications. Likewise, no intergroup differences were observed in long-term follow-up rates pertaining to mortality, recurrence of residual lung infection/TB, occurrence of bronchopleural fistula/empyema, postoperative readmission, or proportions of patients with mMRC ≥ 1 at follow-up. These findings suggest that a preoperative FEV_1_ value of ≤ 2 L should not preclude pneumonectomy, while also highlighting pneumonectomy as a safe and viable treatment option offering favourable short-term and long-term outcomes for DL patients.

In this study, the FEV1 ≤ 2 L group exhibited lower proportions of males and smokers as compared to the FEV1 > 2.0 L group, potentially reflecting common trends of higher FEV1 values and smoking rates among males. The incidence rate of perioperative complications was 26.8%, aligning with findings reported by other research groups [21, 23, 27], thereby demonstrating that most pneumonectomised DL patients recover successfully and resume normal activities after surgery. However, a small number of patients experienced complications, such as recurrent lung infection and bronchopleural fistula, as consistent with results of our previous study [13]. The overall mortality rate at the end of the follow-up period was 7.5%. Interestingly, the incorporation of the actual measured FEV1 as a continuous variable within our regression model revealed no significant correlations between FEV_1_ values and either short-term or long-term postoperative outcomes.

In the 1970s, studies of over 2000 pneumonectomy cases conducted across three research centres demonstrated a mortality rate of 5% for patients with preoperative FEV_1_ values of > 2 L, a clinical standard that continues to be followed [25]. Nonetheless, it should be noted here that lung resection methodologies used to treat DL and lung cancer patients differ. Lung cancer resection typically involves removal of the tumour and surrounding lung tissue with the aim of preserving some respiratory function of the treated lung. Conversely, DL resection involves the excision of irreversibly damaged lung tissue, as confirmed by radiographic imaging [1]. This diseased tissue affects ventilation function within the lesion area, inducing pulmonary-systemic shunting that reduces the ventilation/perfusion ratio, leading to long-term hypoxia. However, organs and tissues of most DL patients gradually adapt to the hypoxic state as the disease progresses [12, 28]. Notably, the excision of non-ventilated tissue in unilateral diffuse lung (DL) patients can notably enhance the ventilation/perfusion ratio, elevate oxygen index values in organs and tissues, and alleviate respiratory distress, ultimately contributing to an improved quality of life [13]. Conversely, lung cancer resection involves eliminating tumor tissue that still retains some gas exchange function. This process significantly reduces the ventilation/perfusion ratio without activating compensatory mechanisms, thereby increasing the risk of stress responses and subsequent postoperative complications [29].

These contrasting outcomes led us to hypothesize that decreased ventilation and the activation of stress responses heighten the probability of postoperative complications in pneumonectomized lung cancer patients compared to those with diffuse lung diseases [30, 31]. Therefore, a preoperative lung function FEV1 > 2 L might be a crucial indicator for pneumonectomy in left/right lung cancer patients but may not hold the same significance for DL patients.

This study had several limitations. First, its retrospective nature and inclusion of a relatively small number of cases may have weakened the robustness of our conclusions. This limitations may stem from low clinician awareness of DL and the absence of established clinical DL treatment guidelines that ultimately hindered our ability to evaluate smaller effect factors. Nevertheless, this study encompassed all DL pneumonectomy cases treated at our centre over a 21-year period, a relatively large sample size as compared to sample sizes of other studies focused on this rare condition. Moreover, as the study was conducted at a single centre, our results might not be generalisable to other healthcare settings or populations. Secondly, a significant proportion of patients (21.7%) were lost to follow-up, potentially affecting the strength of our long-term outcomes data. Thirdly, despite standardised training, variations in expertise among thoracic surgeons over the extended duration of the study and the diverse surgical techniques employed may have introduced biases into our results, particularly those related to post-surgical respiratory failure rates. Finally, our study was solely focused on assessing long-term respiratory distress in DL patients during the follow-up period without analysing lifestyle and psychological factors that could significantly impact respiratory function. Hence, larger-scale clinical studies are warranted to establish specific FEV_1_ assessment and cutoff values for predicting postoperative risks.

## Conclusion

Pneumonectomy appears to be a safe and viable option for DL patients, including those with preoperative lung function FEV_1_ values of ≤ 2 L. No significant differences were observed between DL patients with FEV_1_ values of ≤ 2 L and > 2 L with regard to rates of postoperative survival, complications, recurrence of residual lung infection/TB, bronchopleural fistula, mMRC ≥ 1 at follow-up, and readmission. Hence, the preoperative lung function criterion FEV_1_ > 2 L may not be an appropriate indication for pneumonectomy in DL patients, particularly in the absence of surgical guidelines or consensus. Given the current lack of compelling evidence, further research is warranted to ascertain the true predictive value of FEV_1_ in assessing postoperative risks of DL patients.

## Data Availability

The datasets used and/or analysed during the current study available from the. corresponding author on reasonable request.

## Abbreviations

FEV_1_% pred: forced expiratory volume in one second of predicted
TDL: tuberculosis-destroyed lung
CPA: chronic pulmonary aspergillosis
BPF: bronchopleural fistula
MDR-TB: multidrug-resistant tuberculosis
XDR-TB: extensively drug-resistant tuberculosis
BMI: body mass index
MMV% pred: maximal minute ventilation of predicted
DLCO% pred: lung diffusion capacity for carbon monoxide of predicted
CRP: C-reaction protein
OR: odds ratio
CI: confidence interval
